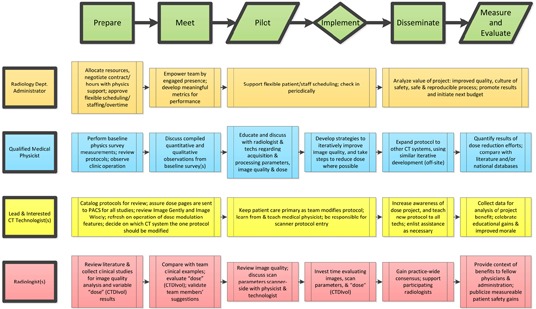# AAPM Medical Physics Practice Guideline 1.a: CT Protocol Management and Review Practice Guideline

**DOI:** 10.1120/jacmp.v14i5.4462

**Published:** 2013-09-06

## Abstract

The American Association of Physicists in Medicine (AAPM) is a nonprofit professional society whose primary purposes are to advance the science, education, and professional practice of medical physics. The AAPM has more than 8,000 members and is the principal organization of medical physicists in the United States.

The AAPM will periodically define new practice guidelines for medical physics practice to help advance the science of medical physics and to improve the quality of service to patients throughout the United States. Existing medical physics practice guidelines will be reviewed for the purpose of revision or renewal, as appropriate, on their fifth anniversary or sooner.

Each medical physics practice guideline represents a policy statement by the AAPM, has undergone a thorough consensus process in which it has been subjected to extensive review, and requires the approval of the Professional Council. The medical physics practice guidelines recognize that the safe and effective use of diagnostic and therapeutic radiology requires specific training, skills, and techniques, as described in each document. Reproduction or modification of the published practice guidelines and technical standards by those entities not providing these services is not authorized.

## 1. Introduction

The review and management of computed tomography (CT) protocols is a facility's ongoing mechanism of ensuring that exams being performed achieve the desired diagnostic image quality at the lowest radiation dose possible while properly exploiting the capabilities of the equipment being used. Therefore, protocol management and review are essential activities in ensuring patient safety and acceptable image quality. These activities have been explicitly identified as essential by several states^1^, ^2^ regulatory and accreditation groups such as the American College of Radiology (ACR) CT Accreditation program,^(3)^ as well as the Joint Commission in its Sentinel Event Alert,^(4)^ among others. The AAPM considers these activities to be essential to any quality assurance (QA) program for CT, and as an ongoing investment in improved quality of patient care.

CT exam protocols are used to obtain the diagnostic image quality required for the exam, while minimizing radiation dose to the patient and ensuring the proper utilization of the scanner features and capabilities. Protocol Review refers to the periodic evaluation of all aspects of CT exam protocols. These parameters include acquisition parameters, patient instructions (e.g., breathing instructions), the administration and amounts of contrast material (intravenous, oral, etc.), and postprocessing parameters. Protocol Management refers to the process of review, implementation, and verification of protocols within a facility's practice.

This is a complex undertaking in the present environment. The challenges in optimization of dose and image quality are compounded by a lack of an automated mechanism to collect and modify protocols system‐wide. The manual labor involved in identifying, recording, and compiling for review and subsequent implementation of all relevant parameters of active protocols is not inconsequential.^(5)^ The clinical community needs effective protocol management tools and efficient methods to replicate protocols across different scanners in order to ensure consistent protocol parameters. The ability to quickly view and understand the myriad of CT protocol parameters contained within a single exam type is critical to the success of protocol review. The ability to quickly identify an outlier protocol parameter would also be hugely beneficial to the CT protocol review process.

This MPPG only applies to CT scanners used for diagnostic imaging. It is not applicable to scanners used exclusively for:

- Therapeutic radiation treatment planning or delivery;
- Only calculating attenuation coefficients for nuclear medicine studies; or
- Image guidance for interventional radiologic procedures.

## 2. Definitions

- CT Protocol ‐ the collection of settings and parameters that fully describe a CT examination^(6)^ Protocols may be relatively simple for some body part specific systems or highly complex for full‐featured, general‐purpose CT systems.^(7)^
- Qualified Medical Physicist ‐as defined by AAPM Professional Policy 1^(8)^

## 3. Staffing Qualifications and Responsibilities

- **The Protocol Review and Management Team**
  Protocol Review and Management requires a team effort; this team must consist of at least a lead CT radiologist, the lead CT technologist, and qualified medical physicist (QMP). In addition, a senior member of the facility administration team should also be involved. This could be the Chief Medical or Administrative Officer for the facility, or a dedicated Radiology Department Administrator/Manager, as determined by hospital leadership. If a senior member of the facility administration team is not a member of the Protocol Review and Management Team, there should be a clear delineation of the reporting structure.
  This team must be responsible for protocol design and review of all parameter settings. Each team member brings different expertise and may have different responsibilities in the Protocol Review and Management process. To be successful, it is very important that the expectations of roles and responsibilities of each member are clearly described. The ability to work together as a team will be an important attribute of each member of this group. The flow chart in Appendix A is an example of how team members should work together and in parallel during the process.^(5)^ Additional examples of protocol management based on one facility's experience are discussed in References 9 and 10. The team members, their qualifications and expectations are described below.
  - **Qualified Medical Physicist (QMP)**
    The first Professional Policy of the AAPM provides a comprehensive definition of a Qualified Medical Physicist (QMP).^(8)^ The subfield of medical physics applicable for CT Protocol Management is Diagnostic Medical Physics. As stated by the Policy, “a [QMP] is an individual who is competent to independently provide clinical professional services in one or more of the subfields of medical physics” and meets each of the following credentials:
    - “Has earned a master's or doctoral degree in physics, medical physics, biophysics, radiological physics, medical health physics, or equivalent disciplines from an accredited college or university; and
    - Has been granted certification in the specific subfield(s) of medical physics with its associated medical health physics aspects by an appropriate national certifying body and abides by the certifying body's requirements for continuing education.”
    - For Diagnostic Medical Physics, the acceptable certifying bodies as of 2012 are: the American Board of Radiology, the American Board of Medical Physics, and the Canadian College of Physicists in Medicine.
  - **Responsibilities of the QMP**
    In the context of CT Protocol Management and Review, the QMP's responsibilities may vary, depending on the type of facility being supported; regardless, the QMP must be involved in the review of all protocols. These considerations **should** be balanced with adequate response times to facility inquiries.
    A QMP's time at a facility **should** include but not be limited to:
    - meeting with the CT Protocol Management and Review team;
    - clinical observation; phantom measurements;
    - side‐by‐side image review with radiologist(s);
    - artifact review with technologist(s) and/or radiologist(s); and
    - discussion of equipment performance and operation, etc.
    While regular dialogue is important, the QMP **should** also remember that facility personnel themselves, in particular the Lead CT Radiologist, **should** lead the CT Protocol Management and Review process; the QMP is an integral member of the team. The QMP may elect to perform baseline dose measurements and image quality tests at the outset of the project, particularly if the QMP does not have personal historical experience with the scanner(s) in the facility.
  - **In‐house QMP**
    For the in‐house QMP, this ongoing CT protocol review project may consume much of his/her time, so the QMP **should** be sure to adequately communicate with his/her supervisor(s), with other team members, and with department/hospital management in this regard. The facility **should** understand that the CT Protocol Management and Review process is an ongoing investment in improved quality of patient care.
    In‐house QMPs may be able to arrange more frequent meetings with CT Protocol Management and Review team members than their consulting colleagues; six to twelve meetings annually may be more appropriate for facilities with in‐house QMPs, with the meeting frequency likely decreasing as time goes on and the facility's protocols are sufficiently improved.
  - **Consulting QMP**
    It is important to note that CT Protocol Management and Review services are above and beyond normal QMPs consulting services (e.g., the annual physics survey), which have traditionally been limited to image quality, dosimetry, and basic protocol review for a few selected examinations. Consultant QMPs **should** make this clear to their clients, and negotiate their services appropriately.
    QMPs providing consulting services **should** maintain regular dialogue with the facility via convenient means (e.g., email, phone, and perhaps text message, if appropriate). It may be beneficial to use a communication process that provides a log of these interactions. It is recommended that the consulting QMP discuss with each facility access to images, including, but not limited to, remote access to the facility's Picture Archiving and Communication System (PACS) for improved consultative capabilities.
    Consulting QMP's **should** work with the facility to arrange mutually agreeable times to visit the facility for CT protocol portfolio review activities. Three to four visits annually may be reasonable.
  - **Qualifications and Expectation of the Lead CT Technologist**
    The American Society of Radiologic Technologists (ASRT) has developed a practice standard entitled *The Practice Standards for Medical Imaging and Radiation Therapy — Computed Tomography Practice Standards*, effective June 19, 2011, which describes the education and certification requirements and scopes of practice for CT technologists.^(11)^
    The Lead CT Technologist is expected to provide the interface between the patient, staff, and the equipment. This includes workflow, the assembly and management of the CT portfolio, and education of the technologist pool.
  - **Qualifications of the CT Radiologist**
    Facilities should refer to the ACR for guidance on the requirements for physicians for accreditation or those in the *Practice Guideline for Performing and Interpreting* ${\text{CJt}}^{12}$) and *CT Accreditation Program Requirements*.^(13)^
    The CT radiologist leads the CT Protocol Management and Review and defines image quality requirements^(14)^

## 4. The Protocol Management Review Process

It is important that the CT Protocol Review and Management team designs and reviews all new or modified protocol settings for existing and new scanners to ensure that both image quality and radiation dose aspects are appropriate. Each member of CT Protocol Management team has a critical role related to his or her specific area of expertise for the evaluation, review, and implementation of protocols. The following elements **should** be considered for inclusion in a specific facilities' protocol review process:

- While performing the review process, the CT Protocol Management team **should** pay particular attention to the oversight and review of existing protocols along with the evaluation and implementation of new and innovative technologies that can improve image quality and/or lower patient dose in comparison to the older protocol.
- Particular attention **should** be paid to the specific capabilities of each individual scanner (e.g., minimum rotation time, automatic exposure controls including both tube current modulation, as well as kV selection technologies, iterative reconstruction, reconstruction algorithms, etc.) to ensure maximum performance of the system is achieved. In addition, consideration **should** be made to consolidate protocols or remove legacy protocols that may not be current or applicable any longer.
- The review process **should** include a review of the most current literature such as ACR practice guidelines,^(12)^ AAPM protocol list,^(7)^ and peer‐reviewed journals, etc., to ensure state‐of‐the‐art protocols are being utilized.

The following considerations are important during review of a protocol:

- **Recommendations for State and National Guidance**
  Local, state, and federal law or regulation varies greatly depending on the state in which the facility is located. The QMP **must** be familiar with applicable federal law and the specific requirements for the state or local jurisdiction where the facility is located. Protocol review and management, while not always explicitly required by state law or regulation, may often facilitate compliance with many provisions within state laws and regulations relating to radiation dose from CT. Links to applicable state regulations can be found at: http://www.aapm.org/government_affairs/licensure/default.asp.
- **Frequency of Review**
  The review process **must** be consistent with federal, state, and local laws and regulations. If there is no specific regulatory requirement, the frequency of protocol review **should** be no less frequent than 24 months. This review **should** include all new protocols added since the last review. However, the best practice would be to review a facility's most frequently used protocols at least annually.
- **Clinically Significant Protocols that Require Annual Review**
  For every facility there are protocols that are used frequently or could result in significant doses. If a facility performs the following six clinical protocols, the CT Protocol Review and Management team **must** review these annually (or more frequently if required by state or local regulatory body). Facilities that do not perform all of the exams listed below **must** select additional protocols at their facility, either the most frequently performed or higher‐dose protocols, to a total of at least six for annual review. The six clinical protocols requiring annual review are:
  - Pediatric Head (1 year old) (if performed at the institution)
  - Pediatric Abdomen (5 year old; 40–50 lb. or approx. 20 kg) (if performed at the institution)
  - Adult Head
  - Adult Abdomen (70 kg)
  - High Resolution Chest
  - Brain Perfusion (if performed at the institution)
- **Protocol Naming**
  A facility should consider naming CT protocols in a manner consistent with the RadLex Playbook ID.^(15)^ This would provide a more consistent experience for patients and referring physicians, and allow more direct comparison among various facilities. This practice may also allow more direct utilization of the ACR Dose Index Registry^(16)^ tools and provide more efficient automated processes with postprocessing workstations. Also, the standardization of protocol names between scanners, even when the scanners are of different makes and models, is strongly encouraged. Appropriate protocol naming will likely result in fewer technologist errors and allow more efficient comparison of protocol parameters between scanners. A facility should consider incorporating version dates in protocol names to easily confirm the latest approved version.
- **Permissions**
  - It is important that each facility establish a process for determining who has permission to access the protocol management systems. Each facility **should** decide and document who has permission to change protocol parameters on the scanner(s). If the scanner allows password protection of protocols, then the facility is encouraged to use this important safety feature. Facilities **should** also decide how passwords are protected and archived.
  - Each facility **should** decide on the process of making protocol adjustments and the frequency with which these adjustments **should** be made. This includes decisions as to what approvals need to be secured before a protocol adjustment may be made, and the documentation process (e.g., a change control log documenting the rationale for each change, as well as who authorized or motivated the change).
  - Each facility **should** consider how to most effectively utilize the NEMA XR 26 standard (Access Controls for Computed Tomography)^(17)^ when these tools become available on scanners at their facility.
- **Acquisition parameters** including kV, mA, rotation time, collimation or detector configuration, pitch, etc., **should** be reviewed to ensure they are appropriate for the diagnostic image quality (noise level, spatial resolution, etc.) necessary for the clinical indication(s) for the protocol, while minimizing radiation dose. For example, a slow rotation time and/or low pitch value would not be appropriate for a chest CT exam due to breath‐hold issues.
  - The facility **should** explicitly review the expected Volume Computed Tomography Dose Index (${\text{CTDI}}_{\text{vol}}$) values. For the limited set of protocols where reference values are available, the ${\text{CTDI}}_{\text{vol}}$ values **should** be compared to the reference values of the ACR CT Accreditation Program,^(3)^ Dose Reference Levels (DRLs),^(18)^ AAPM CT Protocols,^(7)^ or other available reference values for the appropriate protocols.
**Note:** These reference values may be exceeded for individual patient scans (such as for a very large patient, or when the routine protocol is not used because of a different clinical indication, or when the reference value only refers to a single pass in a multipass study).
  - For a facility's routine protocol for a standard sized patient, the expected ${\text{CTDI}}_{\text{vol}}$ values **should** be below these reference values.
- **Reconstruction parameters** such as the width of the reconstructed image (image thickness), distance between two consecutive reconstructed images (reconstruction interval), reconstruction algorithm/kernel/filter, and the use of additional image planes (e.g., sagittal or coronal planes, etc.) **should** also be reviewed to ensure appropriate diagnostic image quality (noise level, spatial resolution, etc.) necessary for the clinical indication(s) for the protocol. For example, a high‐resolution chest exam typically generates thin (~ 1 mm) images using a sharp reconstruction filter.
- **Advanced dose reduction techniques should be considered** when the use of such techniques is consistent with the goals of the exam. Depending on the capabilities of each specific scanner, consider use of the following, if they are available:
  - Automatic exposure control (e.g., tube current modulation or automatic kV selection) methods.
  - Iterative reconstruction techniques.
- **Adjustments of acquisition parameters should be adjusted for patient size**, either through a series of manual adjustments or through the use of automatic techniques (such as tube current modulation methods that adjust for patient size).
- **Radiation dose management tools** fall under two related but different categories, and may provide CT dose data that can be used to determine facility reference dose ranges.
  - Radiation dose management tools that identify when potentially high‐radiation dose scans are being prescribed **should** be implemented when available. This includes dose reporting and tracking software, participation in dose registries, and methods as described in the MITA XR25 standard (“Dose Check”).^(19)^
  - Radiation dose management tools may be used to monitor doses and collect data from routine exams. Statistical analysis of dose parameter values for a specific exam or clinical indication (e.g., average ${\text{CTDI}}_{\text{vol}}$ for a routine noncontrast head) can be provided. Participation in a national registry (such as the ACR Dose Index Registry)^(16)^ and use of commercial dose tracking products are now available for this purpose.
- **Populating Protocols Across Scanners**
  Each facility **should** decide on the process by which protocol parameters are populated across additional scanners (whether this is done manually or by copy/paste, if the scanners allow). The facility **should** decide whether there are ‘master’ or ‘primary’ scanners in the facility where manual protocol adjustments are to be made and archived, and that set of protocols moved to the other similar scanners, or if another strategy will be employed.
- **Documentation**
  The CT Protocol Review and Management team **should** maintain documentation of all changes to protocols, and historical protocols **should** be available for review. Documentation **should** include the rationale for changes (e.g., improve temporal resolution, reduce breath‐hold time, reduce patient dose, etc.). The latest protocol **should** be readily and obviously available to users during clinical protocol selection. In some settings it may be helpful to maintain historical protocols on the scanner, in a less conspicuous location or clearly labeled as a legacy protocol.
  The facility **should** decide and document who is responsible for maintaining the overall protocol description documentation. The facility **should** also describe whether the protocol description documentation is accessible to others for reference, how often it is updated, and how all protocols (on the scanners as well as the protocol description documentation) are archived.
- **Periodic Vendor‐specific Education/Refresher Sessions**
  The CT Protocol Management Process team is responsible for ensuring that each member is adequately trained for protocol review on each scanner used at his or her facility. Each member of the CT Protocol Management Process team should receive refresher training no less than annually or when new technology is introduced that substantially impacts image quality or dose to the patient.
  - Available educational resources **should** be considered in order to keep staff updated on current best practices.
  - Periodic refresher training **should** be scheduled for all members of the CT Protocol Management Process team.
  - Attendance **should** be taken at initial and all refresher‐training sessions, and consequences identified for failure to complete training.
- **Verification**
  Once a CT Protocol Management Process has been established, the CT Protocol Review and Management team **must** institute a regular review process of all protocols to be sure that no unintended changes have been applied that may degrade image quality or unreasonably increase dose.
  As a best practice, the CT Protocol Review and Management team **should** conduct a random survey of specific exam types to verify that the protocols used are acceptable and consistent with protocols specified above. This **should** involve a limited review of recent patient cases to assess:
  - Acquisition and reconstruction parameters,
  - Image quality, and
  - Radiation dose.

## 5. Conclusion

CT protocol management and review is an important part of a CT facility's operation and is considered important by many state regulatory bodies, accrediting, and professional organizations. Protocol parameter control and periodic review will help maintain the facility's image quality to acceptable levels, and will serve to assure patient safety and continuous improvement in the imaging practice.

## ACKNOWLEDGMENTS

This guideline was developed by the Medical Physics Practice Guideline Task Group‐225 of the Professional Council of the AAPM.

TG‐225 Members:

Dianna D. Cody, Chair, PhD, FAAPM

Tyler S. Fisher, MS

Dustin A. Gress, MS

Rick Robert Layman, Jr., MS

Michael F. McNitt‐Gray, PhD, FAAPM

Robert J. Pizzutiello, Jr., MS, FAAPM

Lynne A. Fairobent, AAPM Staff

AAPM Subcommittee on Practice Guidelines – AAPM Committee responsible for sponsoring the draft through the process.

Joann I. Prisciandaro, PhD, Chair

Maria F. Chan, PhD, Vice‐Chair Therapy

Jessica B. Clements, MS

Dianna D. Cody, PhD, FAAPM

Indra J. Das, PhD, FAAPM

Nicholas A. Detorie, PhD, FAAPM

Vladimir Feygelman, PhD

Jonas D. Fontenot, PhD

Luis E. Fong de los Santos, PhD

David P. Gierga, PhD

Kristina E. Huffman, MMSc

David W. Jordan, PhD

Ingrid R. Marshall, PhD

Yildirim D. Mutaf, PhD

Arthur J. Olch, PhD, FAAPM

Robert J. Pizzutiello Jr., MS, FAAPM, FACMP, FACR

Narayan Sahoo, PhD, FAAPM

J. Anthony Seibert, PhD, FAAPM, FACR

S. Jeff Shepard, MS, FAAPM, Vice‐Chair Imaging

Jennifer B. Smilowitz, PhD

James J. VanDamme, MS

Gerald A. White Jr., MS, FAAPM

Ning J. Yue, PhD, FAAPM

Lynne A. Fairobent, AAPM Staff

## APPENDIX

Appendix A: Example of how team members may work together and in parallel during the process.